# Evaluation of the Cytotoxic Effects of Adhesive Systems with Different pH Values on L929 Fibroblast Cells: An In Vitro Study

**DOI:** 10.3390/bioengineering13030338

**Published:** 2026-03-13

**Authors:** Tuba Tunç, Ömer Çellik, Sevgi İrtegün Kandemir, Deniz Evrim Kavak

**Affiliations:** 1 Department of Restorative, Faculty of Dentistry, Dicle University, Diyarbakır 21280, Turkey; dtomercelik@gmail.com; 2 Department of Medical Biology, Faculty of Medicine, Dicle University, Diyarbakır 21280, Turkey; 3 Department of Medical Biology and Genetics, Faculty of Medicine, Dokuz Eylul University, Izmir 35220, Turkey

**Keywords:** cytotoxicity, adhesive systems, biocompatibility, cell viability, MTT assay, L929 fibroblast, ISO 10993-5

## Abstract

Objective: The biocompatibility of adhesive systems is essential for the long-term success of restorative dental procedures due to their close proximity to dentin and pulpal tissues. This study aimed to evaluate the cytotoxic effects of adhesive systems with different pH values on L929 mouse fibroblast cells under in vitro conditions. Materials and Methods: Four commercially available adhesive systems with different pH values—All-Bond Universal, G-Premio Bond, Tokuyama Bond Force II, and Clearfil Universal Bond Quick—were evaluated. Cytotoxicity was assessed using the MTT assay at four different concentrations (0.1%, 0.01%, 0.001%, and 0.0001%) and three incubation periods (24, 48, and 72 h). Cell viability data were analyzed using two-way analysis of variance followed by Bonferroni post hoc tests. Cytotoxicity was interpreted according to ISO 10993-5 criteria. Results: All adhesive systems exhibited concentration-dependent cytotoxicity, with significant reductions in cell viability observed only at the highest concentration (0.1%). At lower concentrations, no cytotoxic effects were detected. Despite having the highest pH value, All-Bond Universal consistently demonstrated the lowest cell viability. In contrast, Tokuyama Bond Force II showed the most favorable cytocompatibility profile, with relatively higher cell viability values over time. Morphological analysis supported the quantitative findings, revealing pronounced cellular alterations at high concentrations and preserved fibroblastic morphology at lower concentrations. Conclusions: adhesive systems demonstrate cytotoxic effects in a concentration-dependent manner, and pH alone is insufficient to predict their biocompatibility. Monomer composition and formulation characteristics appear to play a more critical role in determining cytotoxic behavior. These findings emphasize the importance of appropriate adhesive handling and isolation techniques to minimize tissue exposure and enhance clinical safety.

## 1. Introduction

Adhesive systems play a fundamental role in the clinical success of contemporary restorative dentistry by providing effective bonding between restorative materials and dental hard tissues [1,2]. Inadequate adhesion may lead to marginal microleakage, postoperative sensitivity, secondary caries, and pulpal irritation, ultimately compromising the longevity of restorations [3,4]. Therefore, beyond their mechanical performance, the biocompatibility of adhesive systems is a critical factor that must be carefully evaluated [1,3].

Universal adhesive systems have been widely adopted in clinical practice due to their simplified application protocols, reduced technique sensitivity, and versatility in both self-etch and etch-and-rinse modes [5,6]. Despite these advantages, universal adhesives contain complex formulations composed of functional monomers, solvents, initiators, and stabilizing agents [5]. During clinical application, particularly before complete polymerization, these components may come into direct or indirect contact with dentin, pulp tissue, and surrounding soft tissues, raising concerns regarding their potential cytotoxic effects [7,8]. The acidic pH of universal adhesive systems is a deliberate functional property required for smear layer modification and dentin demineralization through acidic functional monomers [9]. These materials are not intended to persist in an acidic state in vivo, as clinical application involves air-drying, solvent evaporation, and light polymerization, which substantially modify their chemical behavior [10,11,12].

Biocompatibility is defined as the ability of a material to perform its intended function without inducing adverse local or systemic biological responses. In dental materials, cytotoxicity is primarily associated with the release of unreacted monomers and degradation products, which may interfere with cellular metabolism, induce oxidative stress, and impair cell proliferation. In vitro studies have demonstrated that commonly used adhesive monomers, such as Bis-GMA, HEMA, and MDP, may adversely affect fibroblasts and odontoblast-like cells in a concentration- and time-dependent manner.

The acidity (pH) of adhesive systems has been proposed as an important factor influencing cytotoxicity, particularly in self-etch and universal adhesives [13]. Lower pH values may enhance dentin demineralization but have also been associated with increased cellular irritation [14]. Conversely, adhesives with higher pH values are often presumed to be more biologically compatible [15]. However, existing evidence regarding the relationship between adhesive pH and cytotoxicity remains inconsistent, suggesting that pH alone may not adequately predict biological behavior [16,17]. Instead, the interaction between pH, monomer composition, and solvent characteristics may collectively determine cytotoxic outcomes [18].

In vitro cytotoxicity testing using standardized cell culture models represents a widely accepted approach for preliminary biocompatibility assessment of dental materials [19]. The L929 mouse fibroblast cell line, recommended by ISO 10993-5 [20], is commonly used to evaluate cytotoxic responses due to its reproducibility and sensitivity to toxic stimuli [21,22]. Among various viability assays, the MTT assay provides reliable quantitative information on mitochondrial metabolic activity and is frequently employed to assess dose- and time-dependent cytotoxic effects [23,24].

Although several studies have investigated the cytotoxicity of dental adhesives, comparative data focusing specifically on universal adhesive systems with different pH values under standardized conditions remain limited [18,25]. Moreover, the extent to which pH independently influences cytotoxicity, apart from material composition, has not been clearly elucidated [26].

Therefore, the aim of the present study was to evaluate the cytotoxic effects of four commercially available adhesive systems with different pH values on L929 mouse fibroblast cells using an MTT assay. The effects of different concentrations and incubation times were assessed, and cytotoxicity was interpreted according to ISO 10993-5 criteria. This study seeks to clarify whether pH alone is a reliable predictor of cytotoxicity and to contribute to a more comprehensive understanding of the biocompatibility of adhesive systems.

## 2. Materials and Methods

### 2.1. Study Design

This in vitro experimental study was conducted to evaluate the cytotoxic effects of four commercially available adhesive systems with different pH values on L929 mouse fibroblast cells. Cytotoxicity was assessed using the MTT assay at multiple concentrations and incubation periods in accordance with ISO 10993-5 guidelines for biological evaluation of medical devices.

### 2.2. Adhesive Materials

Four adhesive systems commonly used in restorative dentistry were evaluated: All-Bond Universal (Bisco, Schaumburg, IL, USA), G-Premio Bond (GC Europe, Lüven, Belgium), Tokuyama Bond Force II (Tokuyama Dental, Tokyo, Japan), and Clearfil Universal Bond Quick (Kuraray, Houston, TX, USA). The manufacturers, lot numbers, pH values, and main chemical components of the adhesive systems are presented in Table 1.

### 2.3. Preparation of Test Solutions

As the tested adhesive materials were insoluble in cell culture medium, dimethyl sulfoxide (DMSO) was used as a solvent. Stock solutions containing 1% (*w*/*v*) adhesive material were prepared in 10% DMSO. The solutions were sterilized by filtration through 0.2 μm pore-size filters (Millex GP, Merck Millipore, Darmstadt, Germany). Serial dilutions were then prepared using culture medium to obtain final concentrations of 0.1%, 0.01%, 0.001%, and 0.0001%, with the final DMSO concentration maintained below 1% to avoid solvent-related cytotoxic effects.

### 2.4. Cell Culture Conditions

The L929 mouse fibroblast cell line (American Type Culture Collection, ATCC, Manassas, VA, USA), recommended by ISO standards for in vitro cytotoxicity testing, was used in this study. Cells were cultured in Dulbecco’s Modified Eagle Medium (DMEM; Gibco, Waltham, MA, USA) supplemented with 10% fetal bovine serum (FBS), 2 mM L-glutamine, and 100 U/mL penicillin–streptomycin. Cultures were maintained at 37 °C in a humidified incubator with 5% CO_2_. Cell morphology and confluence were routinely monitored using an inverted light microscope (ZEISS Axiovert 135, Oberkochen, Germany).

### 2.5. Cytotoxicity Assessment (MTT Assay)

For cytotoxicity evaluation, L929 cells were seeded into 96-well culture plates at a density of 3 × 10^3^ cells per well and allowed to attach for 24 h. After incubation, the culture medium was replaced with medium containing the adhesive solutions at the specified concentrations. Cells were then incubated for 24, 48, and 72 h. At each time point, 10 μL of MTT solution (5 mg/mL in phosphate-buffered saline) was added to each well, and the plates were incubated for an additional 3 h at 37 °C. Following incubation, the culture medium was carefully removed, and 100 μL of DMSO was added to dissolve the formazan crystals. The plates were gently shaken in the dark for 15 min, and absorbance was measured at 540 nm using a microplate reader (Multiskan Go, Thermo Fisher Scientific, Waltham, MA, USA). Each experimental condition was tested in triplicate wells and repeated in three independent experiments.

### 2.6. Morphological Evaluation

Cell morphology was qualitatively assessed using inverted light microscopy to support the quantitative MTT findings. Representative images were obtained to evaluate changes in cell shape, adhesion, and density following exposure to the adhesive systems at different concentrations and incubation times.

### 2.7. Statistical Analysis

Cell viability values were expressed as percentages relative to the control group. Statistical analyses were performed using SPSS software (version 25.0; IBM Corp., Armonk, NY, USA). Two-way analysis of variance (ANOVA) was used to evaluate the effects of adhesive concentration and incubation time on cell viability. When statistically significant differences were detected, Bonferroni post hoc tests were applied for multiple comparisons. A *p*-value of <0.05 was considered statistically significant.

### 2.8. Ethical Considerations

Ethical approval was not required for this study, as it was conducted entirely in vitro using established cell lines and commercially available materials.

## 3. Results

### 3.1. Effects of Dose and Incubation Time on Cell Viability

The cytotoxic effects of the four adhesive systems on L929 mouse fibroblast cells were evaluated using the MTT assay at four different concentrations (0.1%, 0.01%, 0.001%, and 0.0001%) and three incubation periods (24, 48, and 72 h). The effects of dose, incubation time, and their interaction on cell viability were analysed using two-way ANOVA, and the statistical outcomes are summarized in Table 2.

For All-Bond Universal (ABU) and Tokuyama Bond Force II (TBFII), both dose and incubation time had a statistically significant effect on cell viability (*p* < 0.05), whereas the dose × time interaction was not significant (*p* > 0.05). In contrast, for G-Premio Bond (GPB), both dose and the dose × time interaction showed statistically significant effects on cell viability (*p* < 0.05), while incubation time alone was not significant. For Clearfil Universal Bond Quick (CUBQ), only dose had a statistically significant effect on cell viability (*p* < 0.05), with no significant contribution from incubation time or the dose × time interaction. Across all adhesive systems, dose was identified as the dominant factor influencing cell viability, whereas the effect of incubation time varied depending on the material.

### 3.2. Dose-Dependent Cytotoxicity of Adhesive Systems

A clear dose-dependent reduction in cell viability was observed for all adhesive systems. The lowest cell viability values were consistently recorded at the highest concentration (0.1%), and these reductions were statistically significant compared with the control group (*p* < 0.05).

According to ISO 10993-5 criteria, which define cytotoxicity as a reduction in cell viability greater than 30%, all tested adhesive systems exhibited cytotoxic effects at the 0.1% concentration. In contrast, no cytotoxic effects were detected at lower concentrations (0.01%, 0.001%, and 0.0001%), as cell viability remained above the cytotoxic threshold and did not differ significantly from the control group (*p* > 0.05). These findings indicate that the cytotoxic potential of the tested adhesive systems is strongly concentration-dependent.

### 3.3. Time-Dependent Changes in Cell Viability

Time-dependent changes in cell viability varied among the adhesive systems, particularly at the highest concentration (0.1%). For ABU, GPB, and CUBQ, a progressive decrease in cell viability over time was observed at the highest concentration, with lower viability values at 48 and 72 h compared with 24 h. In contrast, TBFII exhibited a distinct time-related pattern, as cell viability at the highest concentration increased over time. Specifically, higher cell viability values were observed at 48 and 72 h compared with the 24 h time point, indicating a partial recovery of cell metabolic activity with prolonged incubation. At lower concentrations, incubation time did not result in statistically significant changes in cell viability for any of the adhesive systems.

### 3.4. Comparison of Adhesive Systems at Different Time Points

**Figure 1:**
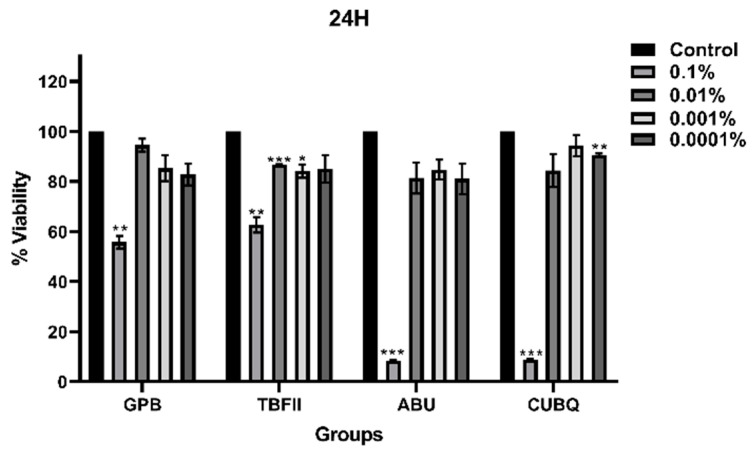
Dose-dependent effects of adhesive systems on L929 cell viability after 24 h. Significance; *: *p* < 0.05, **: *p* < 0.01, ***: *p* < 0.001.

At the 0.1% concentration, statistically significant differences in cell viability were observed among the adhesive systems at all incubation periods. After 24 h of incubation (Figure 1), cell viability differed significantly between materials (*p* < 0.05). Post hoc analysis revealed that GPB and TBFII exhibited significantly higher cell viability than ABU and CUBQ (*p* < 0.05), with ABU showing the lowest viability values at this time point.

**Figure 2:**
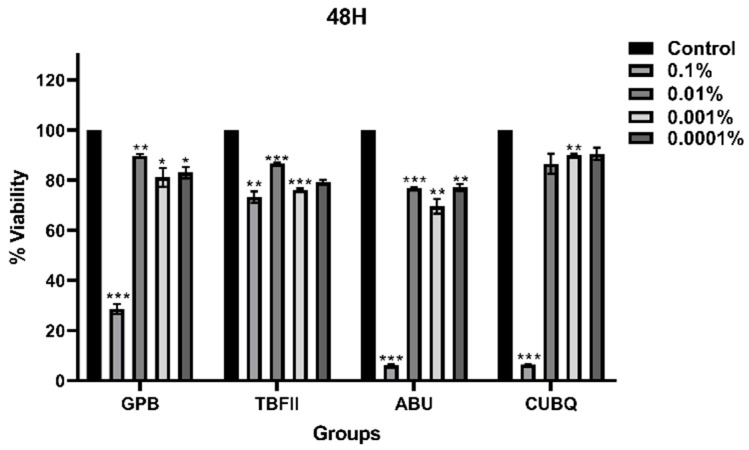
Dose-dependent effects of adhesive systems on L929 cell viability after 48 h. Significance; *: *p* < 0.05, **: *p* < 0.01, ***: *p* < 0.001.

After 48 h of incubation (Figure 2), statistically significant differences were detected among all adhesive systems at all tested concentrations (*p* < 0.05). At the 0.1% concentration, TBFII demonstrated significantly higher cell viability than the other materials (*p* < 0.05). At the lower concentrations, ABU consistently exhibited significantly lower cell viability compared with the other adhesive systems (*p* < 0.05).

**Figure 3:**
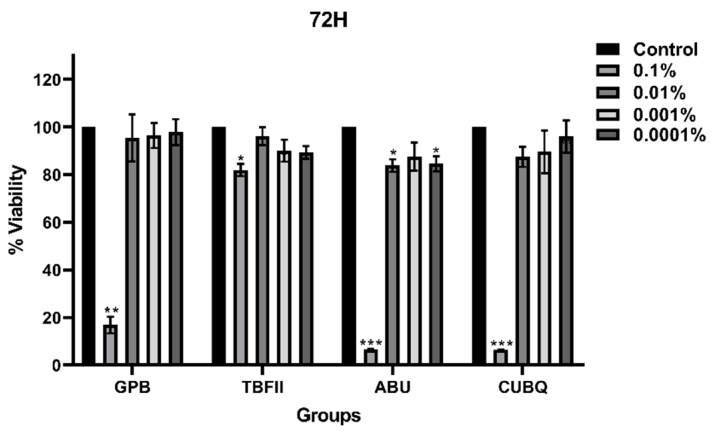
Dose-dependent effects of adhesive systems on L929 cell viability after 72 h. Significance; *: *p* < 0.05, **: *p* < 0.01, ***: *p* < 0.001.

After 72 h of incubation (Figure 3), significant differences among adhesive systems were observed only at the highest concentration (*p* < 0.05). At this concentration, TBFII showed significantly higher cell viability than GPB, ABU, and CUBQ (*p* < 0.05). No statistically significant differences were observed among the materials at lower concentrations.

### 3.5. Morphological Evaluation of L929 Cells

**Figure 4:**
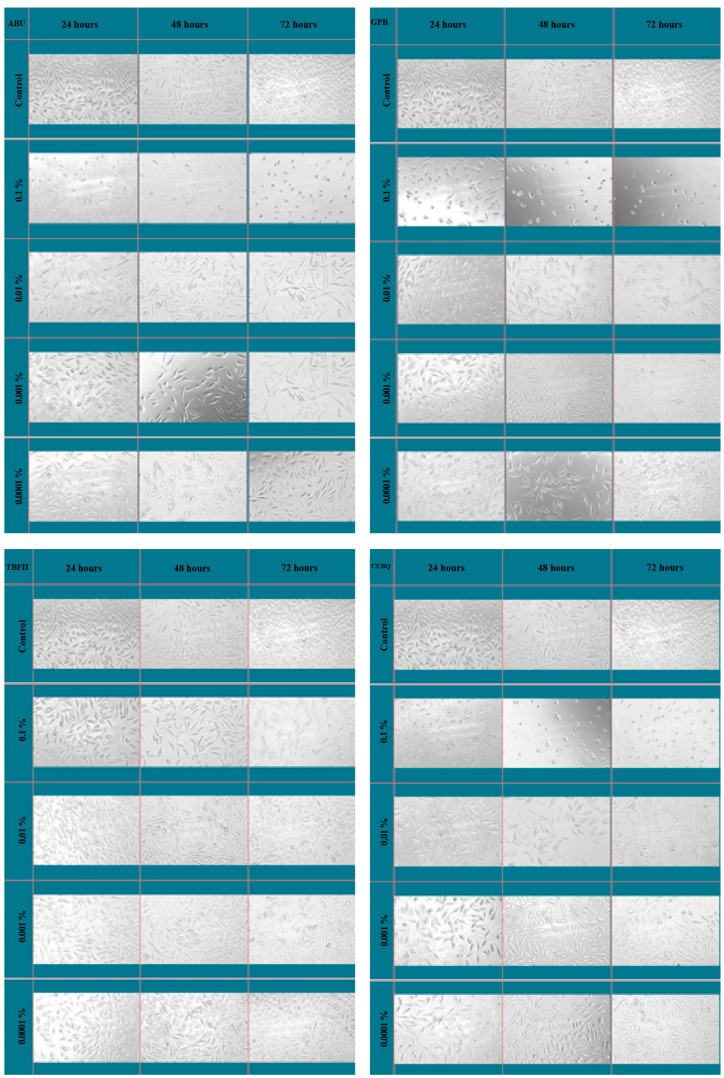
Representative inverted light microscopy images showing morphological changes in L929 mouse fibroblast cells after exposure to adhesive systems at different concentrations and incubation times. Cytotoxic effects at the highest concentration were characterized by cell rounding, loss of spindle-shaped morphology, and reduced cell adhesion, whereas cells treated with lower concentrations exhibited morphology comparable to the control group.

The morphological changes in L929 mouse fibroblast cells following exposure to the adhesive systems were qualitatively evaluated using inverted light microscopy (Figure 4). In the control group, cells exhibited a typical spindle-shaped fibroblastic morphology with intact cytoplasmic extensions and strong adherence to the culture surface. At the highest concentration (0.1%), all adhesive systems induced evident morphological alterations, which became more pronounced with increasing incubation time. These changes included cell rounding, loss of spindle shape, cytoplasmic shrinkage, and partial detachment from the culture surface. A marked reduction in cell density was also observed in these groups compared with the control. Among the tested materials, All-Bond Universal and Clearfil Universal Bond Quick showed more pronounced morphological deterioration, characterized by extensive cell rounding and reduced intercellular connections, particularly at 48 and 72 h. In contrast, Tokuyama Bond Force II-treated cells preserved a relatively higher degree of cellular adherence and structural integrity, even at prolonged incubation times.

At lower concentrations (0.01%, 0.001%, and 0.0001%), the overall cell morphology remained comparable to that of the control group for all adhesive systems, with cells maintaining their fibroblastic shape and normal attachment patterns. No evident morphological signs of cytotoxic damage were observed at these concentrations.

## 4. Discussion

The biocompatibility of adhesive systems is a critical determinant of their clinical reliability, particularly due to their close proximity to dentin and pulpal tissues during restorative procedures [27]. In the present study, the cytotoxic effects of four commonly used adhesive systems with different pH values were evaluated using an in vitro L929 mouse fibroblast model. The findings demonstrated that all tested adhesive systems exhibited concentration-dependent cytotoxicity, with significant reductions in cell viability observed exclusively at the highest concentration (0.1%), while lower concentrations showed no cytotoxic effects according to ISO 10993-5 criteria.

In vitro cytotoxicity assays are widely accepted as a preliminary step for assessing the biological safety of dental materials, as they provide standardized, reproducible, and ethically feasible conditions for evaluating cellular responses [28,29]. Among the available assays, the MTT test remains one of the most commonly employed methods due to its sensitivity in detecting changes in mitochondrial metabolic activity, which closely reflects cell viability [30,31]. Consistent with previous studies, the use of L929 fibroblasts in the present investigation allowed for reliable assessment of the cytotoxic potential of adhesive components that may come into contact with connective tissue-derived cells in clinical settings [21,32,33].

A key finding of this study was that dose was the primary determinant of cytotoxicity, whereas the influence of incubation time varied among materials. This observation aligns with previous reports indicating that the concentration of leachable components, particularly unreacted monomers and solvents, plays a dominant role in determining cellular toxicity [34,35]. At high concentrations, the increased availability of these components may overwhelm cellular defense mechanisms, leading to mitochondrial dysfunction and reduced metabolic activity [36,37]. Interestingly, despite having the highest pH value, All-Bond Universal consistently exhibited the lowest cell viability values among the tested materials. This finding challenges the common assumption that higher pH adhesive systems are inherently less cytotoxic [38]. Previous studies have reported conflicting results regarding the relationship between pH and cytotoxicity, suggesting that pH alone is not a reliable predictor of biological behavior [39]. Instead, the present findings support the notion that monomer composition, solvent type, and their interaction with pH collectively influence cytotoxic outcomes. The presence of monomers such as Bis-GMA and HEMA, which are known to exhibit higher cytotoxic potential, may contribute to the reduced cell viability observed with All-Bond Universal, despite its relatively mild acidity [40]. Although the present study was not designed to isolate the cytotoxic contribution of individual components, the observed differences among the adhesive systems can be plausibly explained by variations in monomer composition and solvent systems [41,42]. Methacrylate-based monomers such as Bis-GMA and HEMA have been widely reported to impair mitochondrial function, induce oxidative stress, and reduce fibroblast viability in a concentration-dependent manner [43]. HEMA, in particular, is known for its low molecular weight and high hydrophilicity, which facilitate cellular diffusion and intracellular accumulation, potentially amplifying cytotoxic effects [44]. Bis-GMA, despite its higher molecular weight, has been associated with mitochondrial dysfunction and apoptotic signaling pathways. In addition, solvent composition may further modulate cytotoxicity by influencing the elution kinetics of residual monomers [45]. Acetone- and ethanol-based systems may differ in their evaporation rates and interaction with aqueous environments, thereby altering the availability of leachable components [46]. Functional acidic monomers such as 10-MDP and 4-MET, while essential for chemical bonding to hydroxyapatite, may also contribute indirectly to cytotoxic responses through their interaction with cellular membranes and local microenvironment [47]. Collectively, these factors suggest that cytotoxicity arises from a complex interplay between monomer composition, solvent characteristics, and concentration, rather than pH alone.

In contrast, Tokuyama Bond Force II demonstrated the most favorable cell viability profile, particularly at prolonged incubation times. Notably, this material showed a partial recovery in cell viability over time at the highest concentration, suggesting a transient cytotoxic effect [48]. This time-dependent improvement may be associated with the volatilization or dilution of cytotoxic components, cellular adaptation mechanisms, or differences in the release kinetics of residual monomers [49]. Similar time-related recovery patterns have been reported in previous in vitro studies evaluating resin-based dental materials [50]. The qualitative morphological analysis further supported the quantitative MTT findings. At the highest concentration, all adhesive systems induced characteristic cytotoxic morphological changes, including cell rounding, loss of spindle-shaped fibroblastic morphology, cytoplasmic shrinkage, and reduced cell adhesion [51]. These alterations are widely recognized indicators of cellular stress and early cytotoxic damage. In agreement with the MTT results, All-Bond Universal and Clearfil Universal Bond Quick produced more pronounced morphological deterioration, whereas cells treated with Tokuyama Bond Force II retained relatively better structural integrity [52,53]. At lower concentrations, the preservation of normal fibroblastic morphology across all materials corroborated the absence of cytotoxicity detected by the MTT assay.

The use of unpolymerized adhesive solutions represents a deliberate worst-case experimental model commonly employed in preliminary in vitro cytotoxicity testing [18]. This approach allows assessment of the intrinsic biological effects of adhesive components prior to polymerization, while acknowledging that clinical procedures such as air-drying and light curing substantially modify material behavior [38]. Moreover, differences in degree of conversion and solvent evaporation efficiency during clinical application may further modulate the elution behavior of adhesive systems and their biological effects [54].

From a clinical perspective, the present findings underscore the importance of minimizing direct and prolonged exposure of pulpal and periapical tissues to unpolymerized adhesive systems. Although in vitro conditions represent a worst-case scenario, they provide valuable insights into the potential biological risks associated with high local concentrations of adhesive components. The observation that cytotoxic effects were limited to the highest concentration supports the clinical relevance of strict isolation techniques, such as rubber dam application, and proper adhesive handling to reduce tissue exposure.

Several limitations of this study should be acknowledged. The evaluation was conducted exclusively under in vitro conditions using a single cell line and a short observation period of up to 72 h. While this approach allows controlled assessment of cytotoxicity, it does not fully replicate the complex biological environment of the oral cavity, where factors such as dentin buffering capacity, pulpal blood flow, immune responses, and long-term material degradation may modulate tissue reactions. The pH of the diluted test solutions was not directly measured; therefore, potential changes in acidity following serial dilution and buffering by the culture medium could not be quantitatively assessed. Furthermore, the absence of polymerization in the experimental design, although intentional to simulate a worst-case exposure scenario, does not reflect routine clinical practice and may overestimate cytotoxic potential.

## 5. Conclusions

Within the limitations of this in vitro study, all tested adhesive systems exhibited concentration-dependent cytotoxic effects, with cytotoxicity observed exclusively at the highest concentration according to ISO 10993-5 criteria. At lower concentrations, none of the materials demonstrated cytotoxic effects on L929 mouse fibroblast cells. Importantly, these findings indicate that the initial pH of adhesive systems alone is not a reliable predictor of cytotoxicity under the present in vitro conditions, as the adhesive system with the highest pH (All-Bond Universal) consistently showed the lowest cell viability. This highlights the critical role of monomer composition and formulation characteristics, rather than acidity alone, in determining the biological behavior of adhesive systems. Among the evaluated materials, Tokuyama Bond Force II demonstrated the most favorable cytocompatibility profile, supported by both quantitative cell viability data and qualitative morphological observations. From a clinical perspective, these results emphasize the importance of proper adhesive handling, effective isolation techniques, and minimizing tissue exposure to unpolymerized adhesive components to enhance biological safety during restorative procedures.

## Figures and Tables

**Table 1:** Adhesives and their contents used in the study.

Adhesive Agent	Manufacturer	Lot Number	Chemical Structure
ABU (pH = 3.2)	Bisco Inc., Schaumburg, IL, USA	2400003696	MDP, bis-GMA, HEMA, ethanol, water, initiator
TBFII (pH = 2.8)	Tokuyama Dental, Tokyo, Japan	3202	HEMA, Bis-GMA, camphor quinone, water, alcohol
CUBQ) (pH = 2.3)	Clearfil Kuraray, Houston, TX, USA	210446	Bis-GMA, HEMA, MDP, ethanol, water, silane
GPB (pH = 1.5)	GC Europe, Lüven, Belgium	2404170	MDP, 4-MET, acetone, dimethacrylate, acetone, water, silane

ABU: All-Bond Universal; TBFII: Tokuyama Bond Force II; CUBQ: Clearfil Universal Bond Quick; GPB: G Premio Bond.

**Table 2:** Statistical values for the dose, incubation and dose × incubation interactions of adhesive materials.

Materials		Df	SS	F	*p*
ABU	Time × Dose	8	245,077	1088	0.441
	Time	2	243.353	4.322	0.044
	Dose	4	31,209.255	1403.212	0.000
	Error (Residual)	10	281.545		
CUBQ	Time × Dose	8	45.808	0.093	0.999
	Time	2	12.147	0.099	0.907
	Dose	4	35,587.337	461.950	0.000
	Error (Residual)	10	615.156		
GPB	Time × Dose	8	2002.338	4.282	0.018
	Time	2	303.696	2.598	0.123
	Dose	4	17,373.776	200.042	0.000
	Error (Residual)	10	584,491		
TBFII	Time × Dose	8	351.377	2.420	0.096
	Time	2	443.487	12.216	0.002
	Dose	4	2390.672	133.067	0.000
	Error (Residual)	10	181.522		

## Data Availability

The datasets generated and/or analyzed during the current study are available from the corresponding author upon reasonable request due to privacy.
